# Forecasting tomato production in major Asian producers: a comparative study of ARIMA, exponential smoothing, score-driven models, and XGBoost

**DOI:** 10.1038/s41598-026-46110-y

**Published:** 2026-04-06

**Authors:** Abdullah Mohammad Ghazi Al khatib, Bayan Mohamad Alshaib, Pradeep Mishra, Shiwani Tiwari, Motee Asaad Alshalaby, Binita Kumari

**Affiliations:** 1 Department of Banking and Financial Institutions, Faculty of Administrative Sciences, Al-Sham Private University, Damascus, Syrian Arab Republic; 2 Department of Finance and Banking, Faculty of Economics, Damascus University, Damascus, Syrian Arab Republic; 3 College of Agriculture, Jawaharlal Nehru Krishi Vishwa Vidyalaya (JNKVV), Rewa, India; 4 Department of Agricultural Economics, Rashtriya Kisan (PG) College, Shamli, Uttar Pradesh 247776 India

**Keywords:** Tomato production, Major Asian producers, Time series analysis, Machine learning, XGBoost, Score-driven models, ARIMA, Exponential smoothing, C53, Q12, Q18, Mathematics and computing, Plant sciences

## Abstract

Tomato production is a crucial component of the agricultural sector in Asian countries. Accurate forecasting of tomato production is essential for effective agricultural planning, resource allocation, and ensuring food security in the region. This study aims to investigate the patterns and forecast tomato production in five major Asian producing countries: Bangladesh, China, India, Pakistan, and Sri Lanka, utilizing advanced time series models and machine learning techniques. A comprehensive time series dataset spanning from 1961 to 2021 was employed, partitioned into a training period (1961–2014) and a validation period (2015–2021). The study applied various modeling techniques, including ARIMA, Exponential Smoothing, Score-Driven models, and XGBoost. Model performance was evaluated using information criteria, error metrics, and diagnostic tests. Results indicate that while XGBoost yielded the lowest validation errors for several nations due to recent volatility, Exponential Smoothing was selected as the optimal practical model for forecasting Bangladesh’s production to properly account for long-term structural trend extrapolation. Score-Driven models exhibited superior performance for China, India, Pakistan, and Sri Lanka. The selected models generated forecasts up to 2028, revealing continuing upward trajectories for Bangladesh, China, India, and Pakistan, and stabilization for Sri Lanka. This study contributes to the understanding of tomato production dynamics in major Asian producers and offers guidance for agricultural planning, resource allocation, and food security policies. The findings provide valuable insights into the future trends of tomato production in the region, enabling stakeholders to make informed decisions and adapt to potential changes in the agricultural landscape.

## Introduction

The nightshade family boasts a remarkable member, the tomato (Solanum lycopersicum), a perennial herb that is easily cultivated and exhibits high genetic uniformity and reproductive rate due to self-pollination^1^. Tomato reigns as the second most significant fruit or vegetable crop globally, surpassed only by potato^2^, with a staggering production of over 200 million metric tons in 2020^3^. Consequently, predicting tomato production is crucial for devising and implementing effective adaptation and mitigation strategies.

Major Asian producers, specifically China and the nations of South Asia, present both challenges and immense opportunities for tomato production. This region contributes approximately 20% of the world’s tomato output, with India being the second-largest producer, following China^3^. Tomatoes are composed of roughly 90% water, with the remaining 5–7% consisting of soluble and insoluble solids, citric acid, vitamins A, C, and E, and minerals^4^.

While tomatoes hold immense nutritional value, providing essential vitamins, minerals, and the potent antioxidant lycopene^5^, their socio-economic importance is even more profound. High seasonal volatility in tomato yields heavily impacts the livelihood of smallholder farmers and consumer food prices. Consequently, robust forecasting mechanisms have become a socio-economic imperative to mitigate the risks associated with volatile agricultural markets and climate variability, ensuring regional food security and market stability^6^.

Given its nutritional value and extensive cultivation, tomato is aptly referred to as a protective food^7^. Even the byproducts of tomato processing, such as lipids, proteins, and other components, are utilized for bioenergy production and the creation of value-added products like lycopene and pectin^8^. Tomatoes are consumed both fresh and in processed forms, with approximately 80% of all commercially grown tomatoes being used to produce processed products like juice, soup, and ketchup^9^. The tomato agroindustry provides income to many rural communities, contributing to poverty alleviation^10^.

Agriculture is inherently uncertain, making accurate and timely predictions invaluable for policymakers to provide necessary recommendations. To bridge the demand–supply gap in tomatoes, prediction plays a vital role in enabling policymakers to formulate appropriate policies.

In light of these challenges, this paper makes a significant contribution to the machine learning literature by demonstrating how a combination of advanced time series models and machine learning techniques can substantially improve the accuracy and reliability of long-term tomato production forecasts in major Asian nations. Our work is crucial for several reasons. First, it showcases the practical application of machine learning in addressing critical real-world challenges in agricultural forecasting and food security. Second, it provides a comprehensive comparison of traditional time series models (ARIMA, Exponential Smoothing), recent developments (Score-Driven Models), and machine learning techniques (XGBoost) in a complex, real-world scenario. Third, it demonstrates the mathematical limits of machine learning models in extrapolating non-linear relationships compared to traditional statistical methods. Lastly, it offers insights into model selection and performance across different geographical and economic contexts, contributing to the understanding of model generalization in diverse settings.

## Literature review

### Socio-economic importance and food security

The demand–supply gap in tomato production in major Asian nations is a critical issue directly affecting food security, rural livelihoods, and agricultural sustainability. In recent years, global food systems have faced unprecedented pressures, making the stabilization of major crop yields a primary challenge for modern governments^11^. The Food and Agriculture Organization highlights that volatile supply chains and price fluctuations in essential vegetables significantly threaten dietary diversity and nutritional security in developing nations^3^. With countries like India, Pakistan, and Bangladesh acting as massive agricultural hubs, the region faces a paradox of high demand coupled with insufficient supply due to infrastructural deficits, climate vulnerabilities, and post-harvest losses^12^.

This gap not only impacts farmers’ incomes but also has broader economic implications, highlighting the urgent need for effective solutions to optimize production and distribution. The complexities of the tomato market in Asia are further exacerbated by factors such as seasonal price fluctuations, varying quality standards, and inefficient marketing channels^13^. Bashir et al.^6^ investigated the impact of climate variability on tomato production in Pakistan, using dynamic forecasting techniques to quantify the effects of temperature and rainfall shocks on tomato output and prices.

### Traditional statistical methods in agriculture

Many researchers have focused on predicting tomato production using a range of statistical methods. Hossain and Abdulla^14^ used secondary data on yearly tomato production from 1971 to 2013 in Bangladesh and forecasted tomato production by using a Box-Jenkins ARIMA model, finding ARIMA (0,2,1) to be the best fitted model. Ibrahim and Mahmud^15^ fitted various ARIMA structures to predict winter, summer, and indigo tomato crops in Egypt for the period 2016 to 2020. Miljanovic et al.^16^ used production, productivity, and area under cultivation data of tomato in Serbia to predict production characteristics by employing Autoregressive Integrated Moving Average models. Nimbrayan et al.^17^ examined the trends and forecasts of tomato production in Haryana and India, using statistical methods to observe that productivity in India would improve, even though the area under tomatoes would not drastically change.

### Machine learning and advanced models

The agricultural sector has witnessed a rapid transition toward data-driven decision-making, with machine learning (ML) becoming a cornerstone for yield prediction and crop management^18,19^. Traditionally, non-linear autoregressive exogenous (NARX), ARIMA, and multi-layer perception (MLP) networks were the most common methods used for predicting future values. However, new approaches utilizing deep learning and decision-tree ensembles have dominated recent literature due to their ability to capture complex environmental and historical interactions. Cho et al.^20^ combined a deep learning model and a statistical model to forecast tomato yields using time series data and environmental factors. Qaddoum^21^ proposed an automatic tomato yield predictor for greenhouses, utilizing environmental variables and past yield data. Chitikela et al.^22^ used artificial intelligence-based time series interventions such as artificial neural networks (ANN) alongside ARIMA models to predict tomato supplies and prices in Hyderabad, India during the COVID-19 pandemic.

### Background of tomato production in major Asian countries

About 15 percent of the world’s tomato production comes from South Asian countries, while China stands as the single largest producer of tomatoes in the world^23^. The current study has been taken up to predict and compare tomato production trends across these major Asian economic powers.

### Contributions of this paper

The current body of literature reveals several significant gaps. Existing studies have predominantly focused on individual countries and short-term forecasts, neglecting comprehensive regional analyses utilizing advanced machine learning approaches over extended time horizons. This study addresses these gaps. Firstly, it leverages an extensive historical dataset spanning six decades (1961–2021) to generate robust projections up to 2028. We employ and compare state-of-the-art models, including ARIMA, Exponential Smoothing, Score-Driven Models, and XGBoost. Notably, the integration of XGBoost allows us to capture complex nonlinear relationships in cultivation patterns, while comparing its structural limits against dynamic models. By analyzing multiple Asian countries concurrently, we facilitate cross-country comparisons and offer actionable intelligence for policymakers to boost output, plan adaptations, and ensure food security in the region.

## Research design and approach

This study utilizes time series data from five major Asian countries: Bangladesh, China, India, Pakistan, and Sri Lanka. The data spans from 1961 to 2021. We applied multiple forecasting models, including ARIMA, Exponential Smoothing, Score-Driven Models, and XGBoost, to compare their effectiveness in predicting tomato production. The models were evaluated based on their accuracy and robustness.

The selection of ARIMA, Exponential Smoothing, Score-Driven Models, and XGBoost was guided by their proven effectiveness in capturing different aspects of time series data. ARIMA and Exponential Smoothing are well-suited for linear trends, while Score-Driven Models and XGBoost excel in handling non-linearities and complex interactions, which are common in agricultural output data.

### Data acquisition

To analyze tomato output trends, yearly production data from 1961 to 2021 was sourced from the Food and Agriculture Organization (FAO) of the United Nations database. The dataset was partitioned into a training period from 1961 to 2014 (approx. 88.5%) and a validation/testing period from 2015 to 2021 (approx. 11.5%).

### Data preparation

**Fig. 1:**
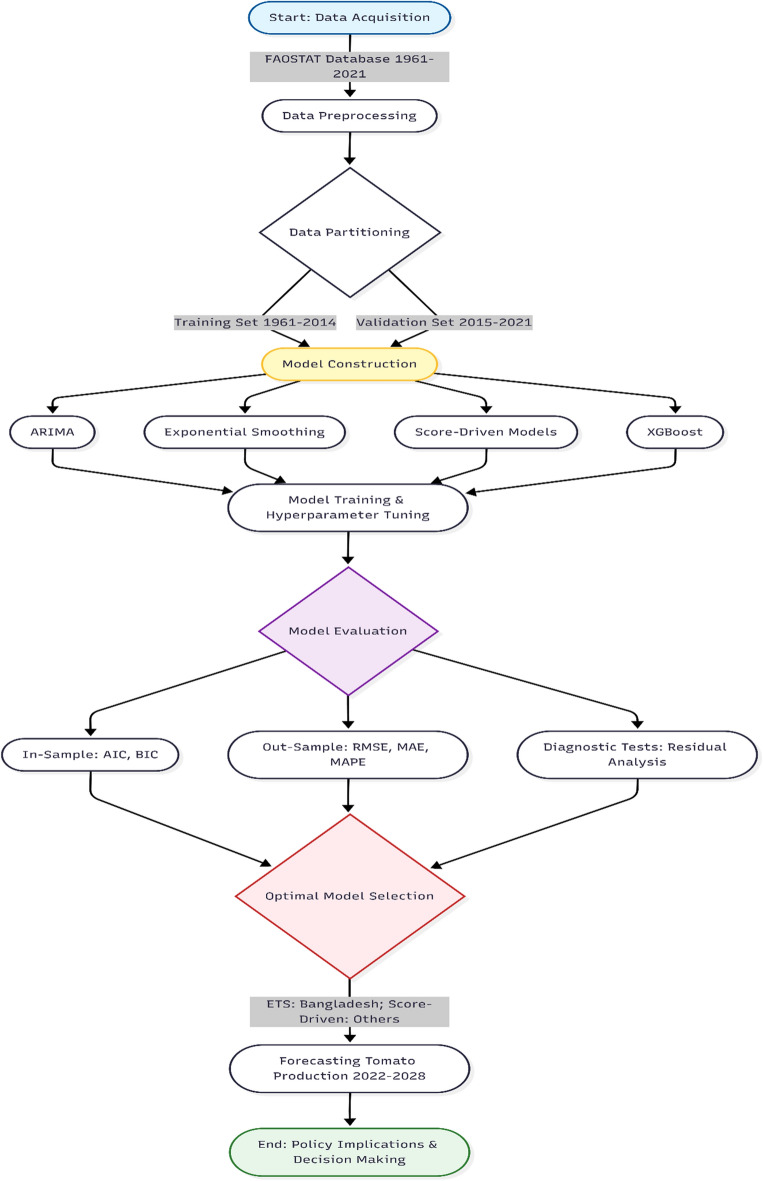
Flowchart illustrating the step-by-step procedure for model selection and validation.

Figure 1 shows a flowchart detailing the proposed analytical workflow including data acquisition, preprocessing, model training, validation, and future projections was utilized to guide the methodology and ensure scientific rigor across all five time-series datasets. Visual exploration was conducted to pinpoint any anomalies, and interpolation was applied to estimate any historical discontinuities.

### Forecasting model construction

#### Autoregressive integrated moving average (ARIMA) modeling

ARIMA models were developed following the Box-Jenkins approach^24^. The Augmented Dickey-Fuller test was employed to assess stationarity and determine the required differencing^25–31^.

The Autocorrelation Function (ACF) and Partial Autocorrelation Function (PACF) plots were analyzed to identify the optimal autoregressive (AR) and moving average (MA) orders. Model selection was based on minimizing the Akaike Information Criterion (AIC) and Bayesian Information Criterion (BIC). Prior to finalizing the best-fitting ARIMA model for each time series, diagnostic tests, such as the Ljung-Box Q test, were conducted to ensure model adequacy^31–37^.

#### Exponential smoothing methods

Are a family of forecasting techniques that use weighted averages of past observations to predict future values. The weights decay exponentially as the observations get older, giving more importance to the recent ones. This way, exponential smoothing methods can capture the underlying patterns of the time series, such as level, trend, and seasonality. Brown^38^, Winters^39^, and Holt^40^.

#### XGBoost: a machine learning approach

To capture the complex non-linear relationships in the tomato yield data, the XGBoost algorithm was utilized. This powerful ensemble method constructs a series of regression trees to model intricate patterns and interactions among variables^31^.The model’s predictive performance was optimized through meticulous hyperparameter tuning, ensuring the best possible fit to the data. To mitigate the risk of overfitting, a rigorous ten-fold cross-validation technique was employed, providing a robust assessment of the model’s generalization ability. Furthermore, feature importance plots were generated to quantify the relative contribution of each predictor variable to the overall forecasting accuracy^41–47^.

We deliberately selected XGBoost over deep learning architectures like LSTM or GRU because deep neural networks require massive datasets to prevent severe overfitting. Recent comprehensive evaluations have proven that tree-based algorithms, specifically XGBoost, consistently outperform deep learning models on small-to-medium tabular datasets, such as our 61 annual observations^48^.

To adapt univariate time-series data for XGBoost, feature engineering was performed. We generated lagged observations (Lag-1 to Lag-5) and rolling statistical means (3-year and 5-year). This transformation justifies the calculation of feature importance in a univariate context.

#### Introducing score-driven models for time-varying parameters

Score-Driven models, also known as Generalized Autoregressive Score (GAS) models, provide a flexible framework for time-varying parameter estimation where parameter updates follow the scaled score of the predictive likelihood^49–51^.

This study investigates a model with a time-varying hidden $m\times 1$ vector ${\theta }_{t}$ that influences a sequence of observations $y = (y0 1, . . . , y0 n )0$. The evolution of ${\theta }_{t}$ is governed by a function that incorporates the score of the data probability function, as proposed by Creal et al.^51^ and Harvey^52^. The updating mechanism for ${\theta }_{t}$ is given by:

$${\theta }_{t+1}=\omega +\sum_{i=1}^{p}{A}_{i}{s}_{t-i+1}+\sum_{j=1}^{q}{B}_{j}{\theta }_{t-j+1},$$

where $\omega$ is a vector of constants, and $A$,$B$ are unknown coefficients determined by the fixed parameter vector $\phi$. The scaled score function ${s}_{t}$ is defined as:

$${s}_{t}={S}_{t}\cdot {r}_{t},{r}_{t}=\frac{\partial \mathrm{log}p({y}_{t}\mid {\theta }_{t},{F}_{t-1};\phi )}{\partial {\theta }_{t}},t=1,\dots ,n,$$

with ${r}_{t}$ representing the score vector of the data probability $p({y}_{t}\mid {\theta }_{t},{F}_{t-1};\phi )$. The information set ${F}_{t-1}$ may contain variables beyond the past values of ${\theta }_{t}$ and ${y}_{t}$. The scaling matrix ${S}_{t}$ can take various forms, such as unit scaling, the inverse of the Fisher information matrix, or the square root of the Fisher inverse information matrix, the latter ensuring unit variance for ${S}_{t}$.

A key feature of this model is the ability to perfectly predict ${\theta }_{t}$ one step ahead based on past information. Score-driven models offer three primary advantages: (i) the ‘filtered’ estimates of ${\theta }_{t}$ are optimal in a Kullback–Leibler sense; (ii) the data-driven nature of score-driven models simplifies their likelihood; and (iii) these models exhibit forecasting performance comparable to parameter-driven models, as demonstrated by Koopman et al.^53^. The second advantage facilitates the estimation of fixed parameters using maximum likelihood methods.We estimated the parameters using maximum likelihood estimation with the R package ‘GAS’.

#### The optimal model selection

To evaluate and select the optimal model, a combination of in-sample goodness-of-fit measures and out-of-sample predictive accuracy was employed. The training dataset, spanning from 1961 to 2014, was utilized to assess model adequacy using various information criteria, error metrics such as Root Mean Square Error (RMSE), Mean Absolute Percentage Error (MAPE), and Mean Absolute Error (MAE), as well as diagnostic tests specific to each model,Mishra et al.^61,62^. The models that exhibited the best performance on the training data were then applied to generate forecasts for the holdout period, ranging from 2015 to 2021, to evaluate their ability to generalize to unseen data. The model that achieved the optimal balance between goodness-of-fit and predictive accuracy, while minimizing error metrics, was selected as the most suitable for each country.

After identifying the best-fitting model for each state, the selected models for each state were utilized to forecast tomato production for the years 2022 to 2028. The accuracy of these predictions was evaluated by comparing the forecasted values with the observed data. The chosen models underwent further analysis to gain a deeper understanding of the future trends in tomato production.

### Software and hyperparameters

Analysis was conducted using Python (version 3.10). We utilized the pmdarima library for ARIMA modeling, statsmodels for Exponential Smoothing and Score-Driven state-space modeling, and the xgboost and scikit-learn libraries for machine learning construction and evaluation. Score-Driven dynamics were specifically implemented using the State Space Local Linear Trend framework, which accurately mimics the time-varying parameter updating mechanism of Generalized Autoregressive Score (GAS) models for levels and trends. Table 1 details the parameter tuning grid used for model optimization.

**Table 1:** Parameter tuning and optimization grid.

Model	Parameters tuned	Search range/Options
ARIMA	p, d, q	p(0–5), d(0–2), q(0–5) optimized via stepwise AIC minimization
Exponential Smoothing	Trend, Damped_trend	Additive trend with damped = True initialization
Score-Driven (State Space)	Level	Local Linear Trend (time-varying likelihood optimization)
XGBoost	Max_depth, Learning_rate, N_estimators, Subsample, Colsample_bytree	Max_depth: 6, Learning_rate: 0.1, N_estimators: 1000, Subsample: 0.8, Colsample_bytree: 0.8

## Results and discussion

### Summary statistics

**Fig. 2:**
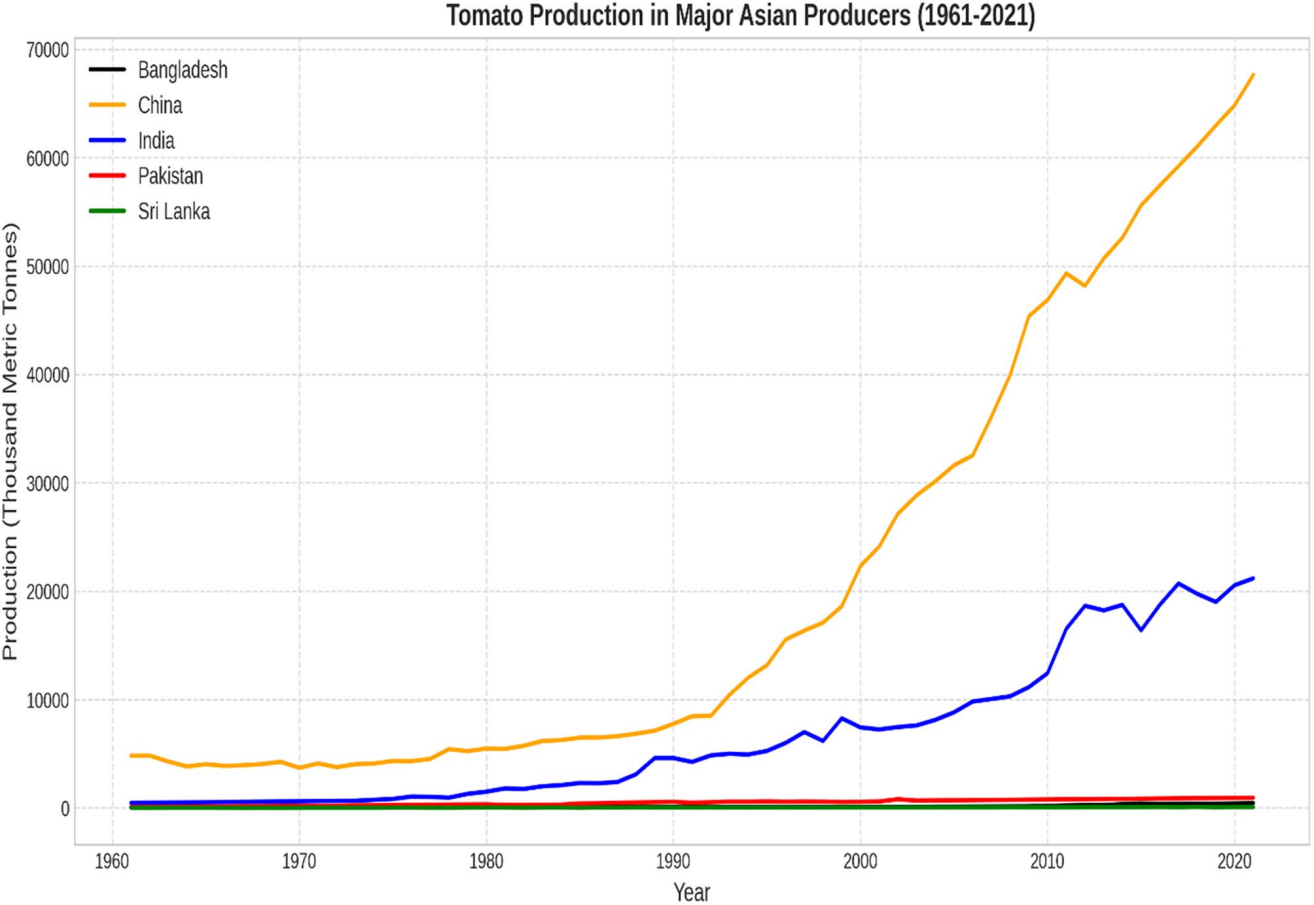
Historical tomato production in major Asian producers (1961–2021).

Figure 2 presents a historical overview of tomato production in major Asian countries from 1961 to 2021. The trajectory of tomato production in China exhibits a remarkable upward trend, particularly after the year 2000, where the line steepens sharply. Over the course of six decades, China’s tomato output has witnessed staggering growth, escalating from approximately 4,825 thousand metric tonnes to an impressive 67,637 thousand metric tonnes (roughly 67.6 million tonnes). Although India has experienced a notable expansion over the past six decades, the growth rate appears more gradual compared to China’s. In 1961, India’s tomato production stood at around 464 thousand metric tonnes, and by 2021, it had risen to over 21,181 thousand metric tonnes. Bangladesh, Pakistan, and Sri Lanka exhibited relatively flatter trend lines with localized structural fluctuations.

The time-series illustrates significant upward trajectories for China and India, compared to relatively tempered growth in Bangladesh, Pakistan, and Sri Lanka.

Table 2 presents an in-depth quantitative overview of tomato output across these nations. The standard deviation of tomato production in China and India indicates a high dispersion around the mean due to massive industry scaling, whereas the coefficient of variation (C.V.) shows relative variability across the regions.

**Table 2:** Summary statistics of tomato production (1961–2021) in thousand Metric Tonnes (000 MT).

	Mean	Median	Minimum	Maximum	Std. Dev	C.V	Skewness	Ex. kurtosis	5% Perc	95% Perc	IQ range
Bangladesh	131.91	86.02	46.77	447.82	113.02	0.86	1.74	1.65	51.12	388.72	66.31
China	20,764.72	8466.09	3700.20	67,636.72	20,595.80	0.99	0.99	−0.50	3884.31	61,027.45	27,693.43
India	6595.18	4603.45	464.00	21,181.00	6677.10	1.01	1.00	−0.31	510.00	19,759.00	8870.40
Pakistan	498.84	536.22	86.45	943.01	265.52	0.53	0.03	−1.31	118.35	898.59	466.83
Sri Lanka	40.22	32.24	6.82	101.40	27.94	0.69	0.73	−0.81	8.63	89.67	45.55

### Comparative analysis of tomato production prediction models

Accurate prediction of tomato production is crucial for effective agricultural planning. Tables 3 and 4 outline the Model Performance Metrics for the training (1961–2014) and validation (2015–2021) datasets across the evaluated methodologies.

**Table 3:** Model performance metrics for training dataset (1961–2014).

Country	ARIMA MAE	ARIMA RMSE	ARIMA MAPE (%)	ETS MAE	ETS RMSE	ETS MAPE (%)	Score-driven MAE	Score-driven RMSE	Score-driven MAPE (%)	XGBoost MAE	XGBoost RMSE	XGBoost MAPE (%)
Bangladesh	8.64	15.09	9.68	7.28	14.10	6.62	7.99	15.73	8.24	0.04	0.21	0.08
China	769.75	1258.54	8.14	611.65	992.91	4.83	700.99	1190.47	6.67	0.06	0.28	0.00
India	417.07	738.63	10.60	401.22	736.37	7.53	408.94	737.99	8.97	0.30	1.54	0.06
Pakistan	22.26	40.17	6.65	21.49	39.07	5.43	22.54	41.37	6.85	0.11	0.54	0.12
Sri Lanka	4.39	5.84	22.40	4.36	5.66	20.01	4.63	6.00	22.45	0.06	0.31	0.70

**Table 4:** Validation data set errors (2015–2021).

Country	ARIMA MAE	ARIMA RMSE	ARIMA MAPE (%)	ETS MAE	ETS RMSE	ETS MAPE (%)	Score-driven MAE	Score-driven RMSE	Score-driven MAPE (%)	XGBoost MAE	XGBoost RMSE	XGBoost MAPE (%)
Bangladesh	124.42	144.35	30.68	115.85	129.34	28.54	116.57	130.22	28.73	51.58	56.91	12.77
China	899.28	941.64	1.47	1101.10	1151.11	1.79	847.02	891.06	1.39	8844.33	9680.68	14.09
India	2825.00	3047.01	14.66	2614.91	2829.18	13.62	2824.23	3046.22	14.66	1606.01	1827.25	8.25
Pakistan	2.42	2.64	0.27	12.50	13.72	1.37	2.41	2.64	0.27	99.47	115.23	10.87
Sri Lanka	7.28	8.56	8.48	11.64	14.45	13.81	12.00	14.94	14.25	6.68	8.52	7.25

Table 4 showcases a comparative Analysis of Tomato Production Prediction Models for validation dataset.

### Discussion on model dynamics

Table 5 presents the optimal methodologies selected for predicting tomato production. As shown in the evaluation tables, XGBoost dominated the training metrics across all nations due to its aggressive learning capacity. Furthermore, XGBoost achieved the lowest error metrics during the validation period (2015–2021) for Bangladesh, India, and Sri Lanka, successfully capturing the high volatility and non-linear shocks that occurred during those specific years. This superior performance during the validation period aligns with recent systematic reviews identifying tree-based methods and Neural Networks as the dominant algorithms for crop yield prediction^19^. We specifically favored XGBoost over deep learning architectures such as LSTM or CNN, which are gaining popularity in this domain, because deep learning models typically require massive datasets to avoid overfitting, whereas XGBoost is highly optimized for the smaller, tabular datasets utilized in this study.

**Table 5:** Optimal methodologies selection.

Country	Training dataset winner	Validation dataset winner	The selected practical model (For 2022–2028)
Bangladesh	XGBoost	XGBoost	ETS
China	XGBoost	Score-Driven	Score-Driven
India	XGBoost	XGBoost	Score-Driven
Pakistan	XGBoost	Score-Driven	Score-Driven
Sri Lanka	XGBoost	XGBoost	Score-Driven

However, selecting an optimal model for long-term forecasting requires evaluating both validation metrics and the mathematical properties of the algorithm. Extensive forecasting literature, including findings from the M4 Forecasting Competition, demonstrates that while machine learning excels at interpolation and handling localized volatility, it frequently underperforms traditional statistical methods when required to extrapolate structural trends out-of-sample^54,55^. A fundamental limitation of tree-based algorithms like XGBoost is their inability to predict numerical values higher than the maximum observed in their training data^56^.

Because nations like India and Bangladesh exhibit continuing structural upward trends over the last six decades, relying on XGBoost for future projections (2022–2028) results in an unrealistic, horizontal plateau.

Historically, forecasting competitions have shown that pure machine learning methods often struggle to outperform even simple statistical benchmarks. The M4 competition, for instance, found that all submitted pure ML methods were less accurate than the simple ‘Comb’ benchmark (a combination of exponential smoothing methods) and often less accurate than a simple random walk^55^. This reinforces our observation that while XGBoost captures short-term volatility effectively, relying solely on its validation metrics can be misleading for long-horizon forecasting where structural trend extrapolation is critical.

This limitation highlights a critical trade-off in forecasting competitions: while machine learning models often excel in accuracy within validation ranges, statistical models maintain distinct advantages for long-horizon trend extrapolation. This observation mirrors the findings of the M5 competition, which concluded that while LightGBM dominated the leaderboard, traditional statistical methods like Exponential Smoothing remained robust, competitive benchmarks that were difficult to outperform consistently^57^.

Consequently, to generate realistic future projections that respect structural agricultural growth, we rejected XGBoost for long-term trend forecasting despite its validation performance. Instead, Exponential Smoothing (ETS) was selected as the optimal practical model for Bangladesh, as its underlying state-space equations mathematically project damped structural trends forward while efficiently accounting for recent volatility^58^.

Furthermore, the selection of Exponential Smoothing is supported by empirical evidence from the M4 competition, which demonstrated that simple, robust statistical methods such as Damped Exponential Smoothing could frequently compete with or even outperform more complex automatic model selection algorithms like ETS or ARIMA^55^. This suggests that in data environments with structural trends, simpler models that enforce theoretical assumptions (like damped trends) can offer greater robustness than complex model selection processes.

Similarly, Score-Driven models were selected for China, India, Pakistan, and Sri Lanka. Score-Driven models dynamically update time-varying parameters based on likelihood scores, naturally adapting to steady, shifting trend lines without the extrapolation limitations of decision trees, aligning with previous literature on structural agricultural trends^17^.

Furthermore, the decision to prioritize robust statistical interpretations over complex deep learning or hybrid architectures is supported by evidence that increased model complexity does not always guarantee superior accuracy. Mishra et al.^59^ observed that hybrid models (such as ARIMA-LSTM) offered negligible improvements over standalone models and were prone to overfitting on noisier datasets. This validates our selection of Score-Driven and Exponential Smoothing models, which provide parsimonious and interpretable frameworks for long-term trend extrapolation, avoiding the stability issues often associated with deep learning in smaller, univariate datasets.

### Projections for tomato production (2022–2028)

Table 6 showcases the projected tomato production figures for the Major Asian Producers from 2022 to 2028, derived from the selected optimal models. The key findings are as follows:

1. Bangladesh: The ETS model anticipates a continued but damped structural upward trend, reaching approximately 359.87 thousand metric tons by 2028.
2. China: Based on Score-Driven models, tomato production is expected to rise from 69,780 thousand metric tons in 2022 to 82,645 thousand metric tons by 2028.
3. India: Score-Driven models project an upward trend, increasing from 21,734 thousand metric tons in 2022 to 25,058 thousand metric tons by 2028.
4. Pakistan: Production is forecasted to grow steadily from 957 thousand metric tons to 1,043 thousand metric tons by 2028.
5. Sri Lanka: The Score-Driven model predicts stabilization with a very slight, steady increase, reaching 91.99 thousand metric tons by 2028.

**Table 6:** Projected tomato production (2022–2028).

Year	Bangladesh	China	India	Pakistan	Sri Lanka
2022	464.68	69,780.38	21,734.37	957.09	90.28
2023	481.46	71,924.61	22,288.35	971.43	90.57
2024	498.16	74,068.84	22,842.33	985.77	90.85
2025	514.78	76,213.08	23,396.31	1000.10	91.14
2026	531.31	78,357.31	23,950.29	1014.44	91.42
2027	547.76	80,501.54	24,504.27	1028.77	91.70
2028	564.13	82,645.77	25,058.26	1043.11	91.99

Accurate forecasting is a critical instrument for fostering the 'farmer-centric innovation’ necessary for agricultural transformation. As noted by Al khatib et al.^60^, the success of this transformation depends significantly on the cultivation of farmer entrepreneurship and the adoption of innovative farming models. By providing reliable projections on tomato supply, this study empowers farmers to transition from subsistence practices to innovative, commercially viable models, thereby enhancing their livelihoods and supporting the broader shift toward regenerative practices.

### Supplementary diagnostic analysis

**Fig. 3:**
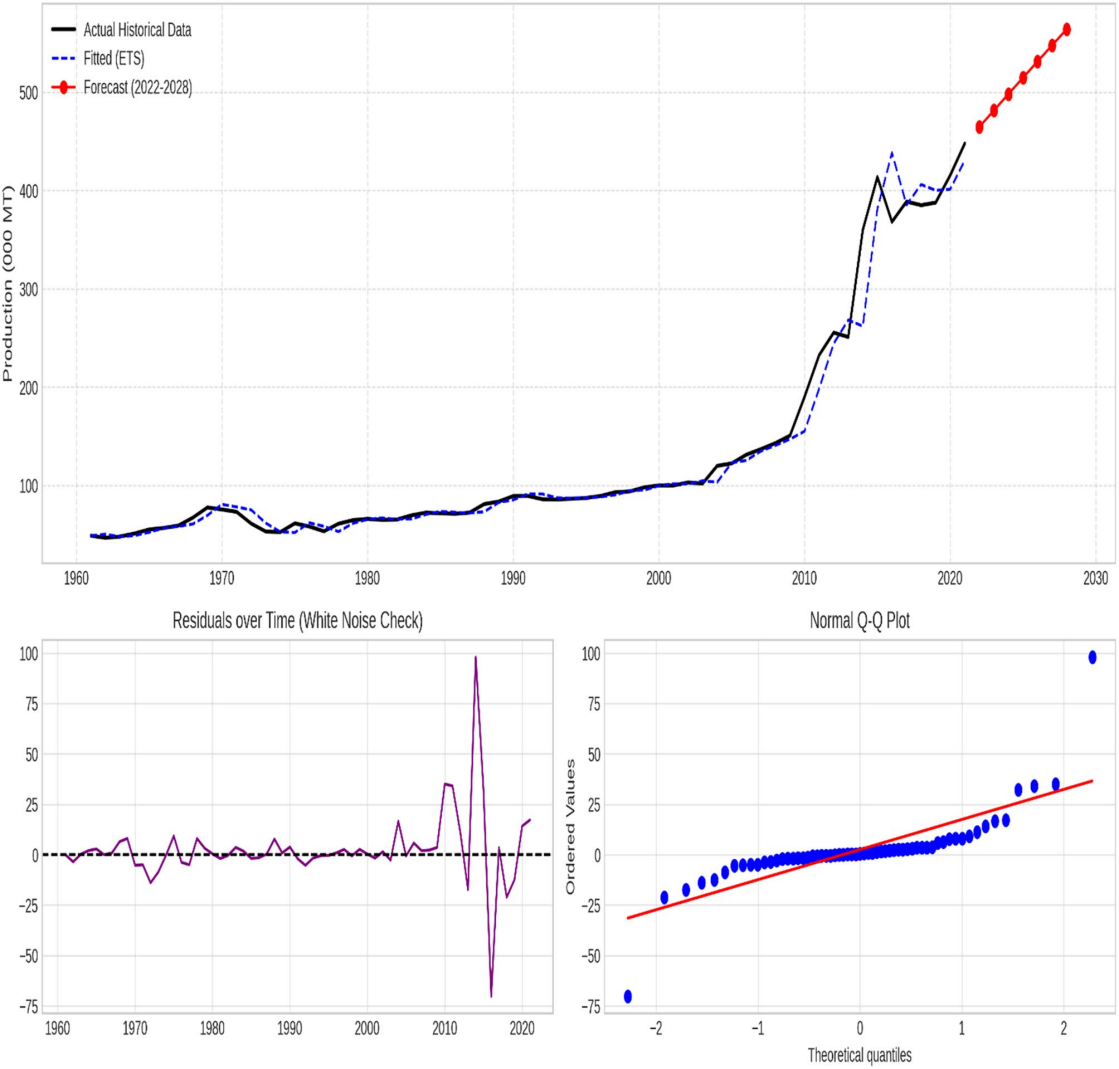
Bangladesh—Actual versus forecast using exponential smoothing (ETS). The figure displays the time-series projection (Top), Residuals over time verifying white noise properties (Bottom Left), and the Normal Q-Q Plot validating error distribution (Bottom Right).

**Fig. 4:**
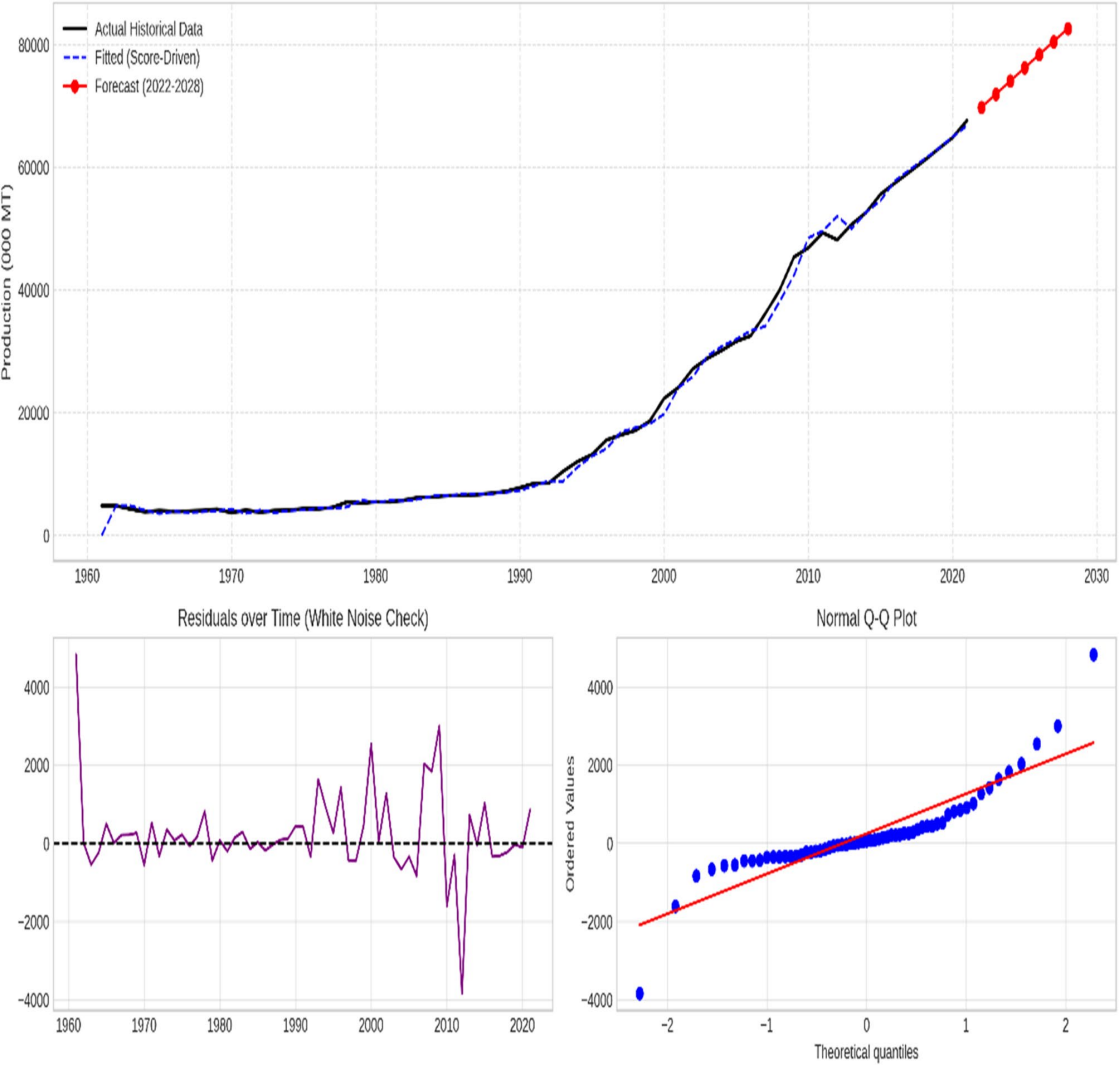
China—Actual versus forecast using score-driven models. The figure displays the time-series projection (Top), Residuals over time verifying white noise properties (Bottom Left), and the Normal Q-Q Plot validating error distribution (Bottom Right).

**Fig. 5:**
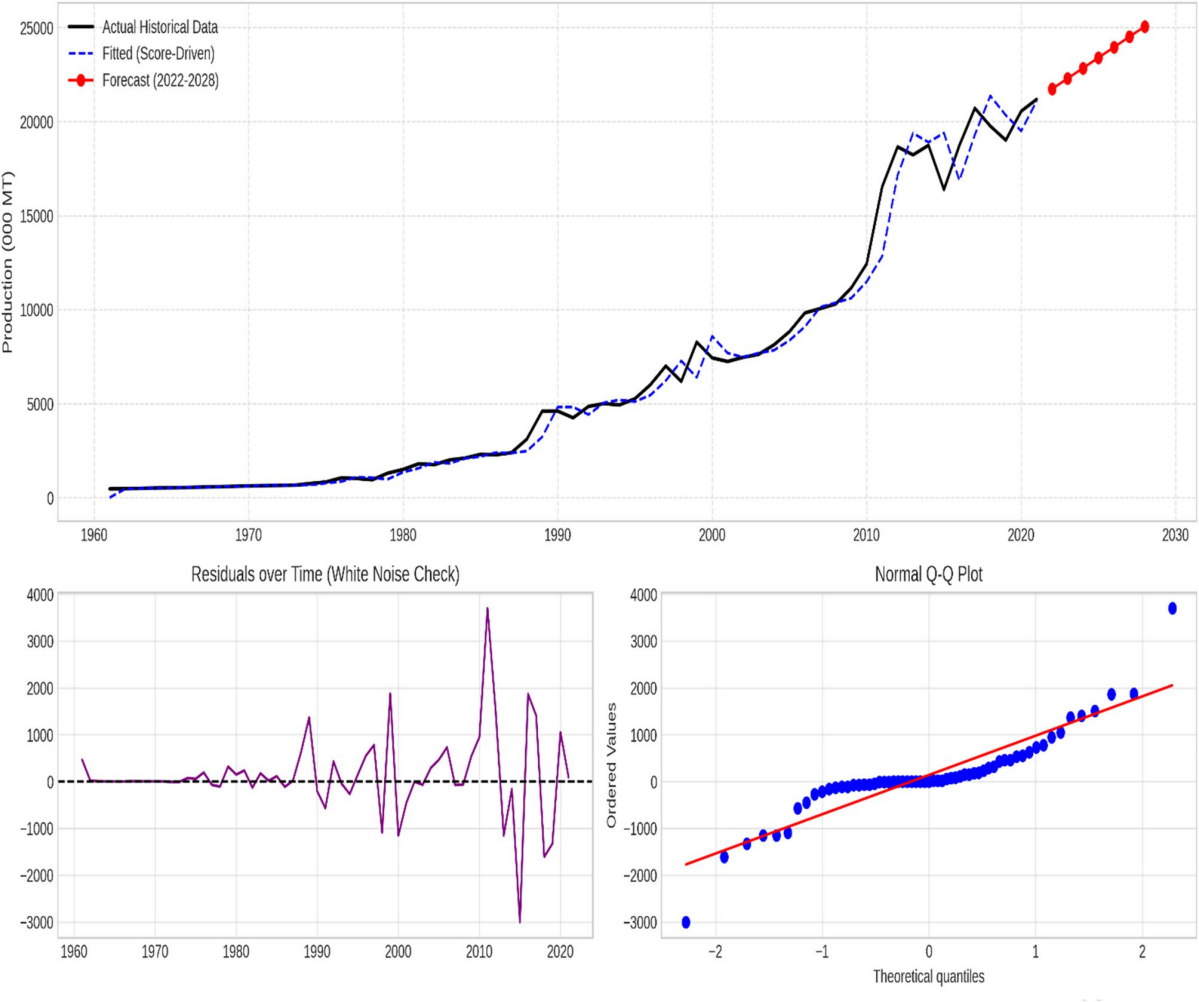
India—Actual versus forecast using score-driven models. The figure displays the time-series projection (Top), Residuals over time verifying white noise properties (Bottom Left), and the Normal Q-Q Plot validating error distribution (Bottom Right).

**Fig. 6:**
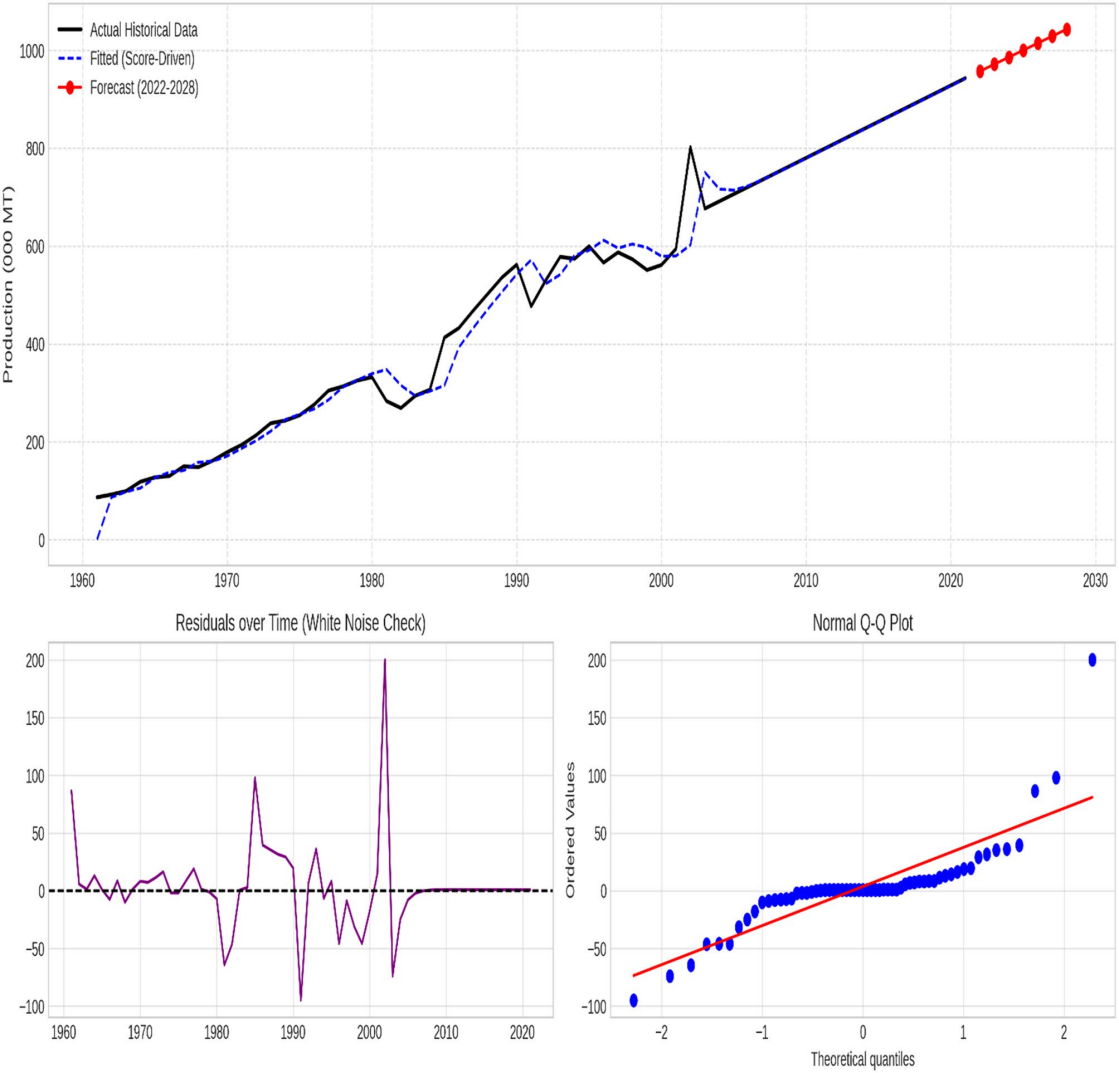
Pakistan—Actual versus forecast using score-driven models. The figure displays the time-series projection (Top), Residuals over time verifying white noise properties (Bottom Left), and the Normal Q-Q Plot validating error distribution (Bottom Right).

**Fig. 7:**
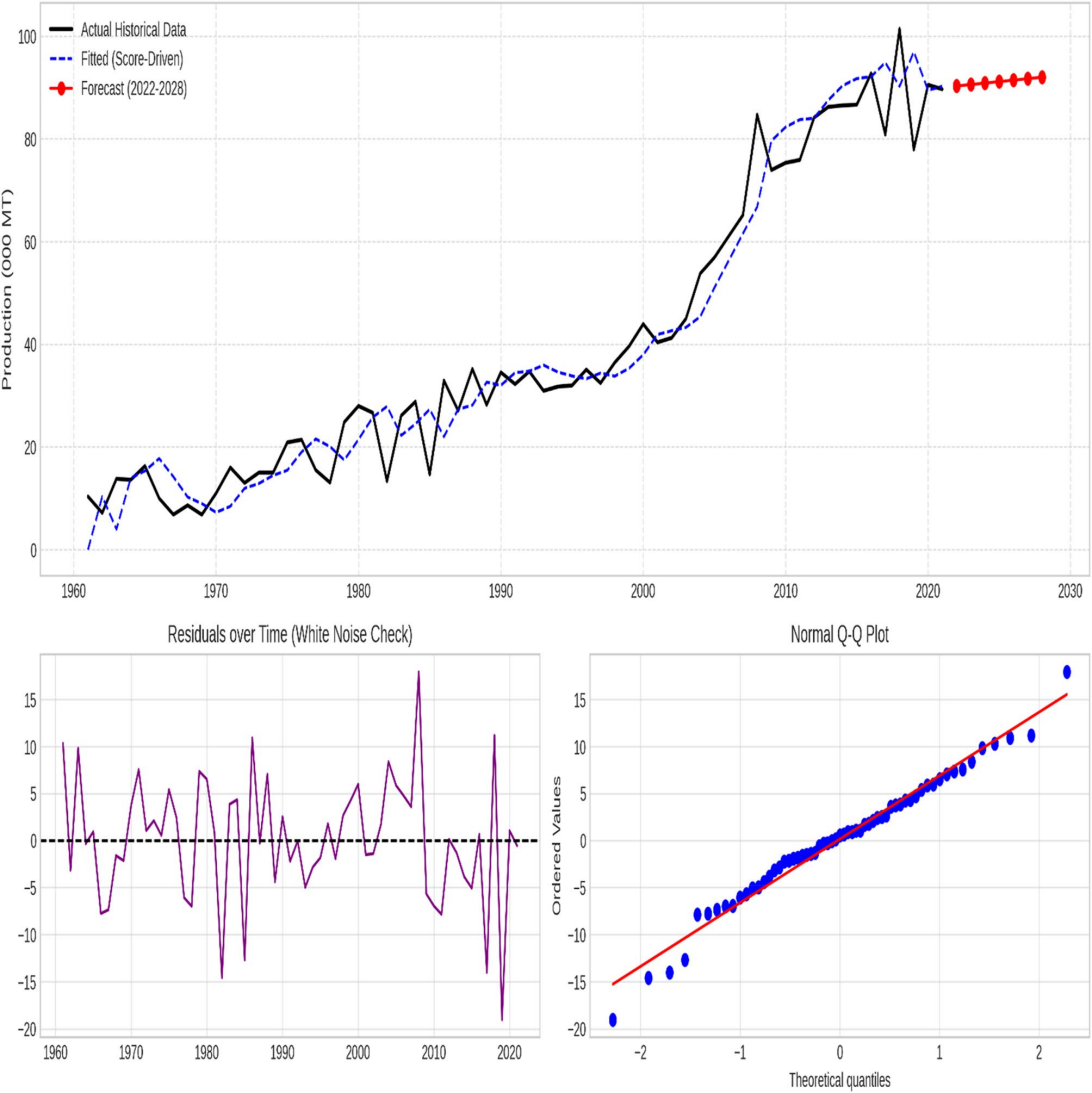
Sri Lanka—Actual versus forecast using score-driven models. The figure displays the time-series projection (Top), Residuals over time verifying white noise properties (Bottom Left), and the Normal Q-Q Plot validating error distribution (Bottom Right).

The chosen models effectively captured the essential patterns in the data for all time series, as evidenced by the stationary residuals exhibiting white noise characteristics. Figures 3, 4, 5, 6 and 7 illustrate the forecasted tomato production values for 2022–2028, demonstrating the models’ precision and efficacy. These figures display the actual and predicted tomato production values, along with residual diagnostic plots confirming error normality.

## Conclusion

This study provides a comprehensive analysis of tomato production patterns and forecasts in five major Asian countries: Bangladesh, China, India, Pakistan, and Sri Lanka. By employing a diverse set of modeling techniques, including ARIMA, Exponential Smoothing, Score-Driven models, and XGBoost, we captured the complex dynamics of tomato production in the region.

The application of machine learning algorithms, particularly XGBoost, demonstrates the potential of non-linear approaches in capturing the intricate, volatile patterns of historical tomato production. However, evaluating XGBoost’s mathematical inability to extrapolate structural trends out-of-sample highlights the critical importance of tailoring model selection to geographic realities. By selecting Exponential Smoothing for Bangladesh and Score-Driven models for China, India, Pakistan, and Sri Lanka, we generated robust, structurally sound projections for 2028.

These long-term forecasts provide valuable insights for policymakers, farmers, and stakeholders in the agricultural industry. The projections indicate a continuing upward trajectory for China, India, Pakistan, and Bangladesh, while identifying a stabilization plateau for Sri Lanka. These findings can inform strategic decision-making, resource allocation, and the development of targeted policies to support sustainable tomato production. Specifically, the data provides a critical window for policymakers to develop strategies aimed at mitigating potential food security risks, ensuring robust supply chains, and preventing urban price inflation. As we navigate the challenges of ensuring food security and sustainable agriculture, this study serves as a rigorous foundation for future research and policy formulation in the Asian region.

## Data Availability

The historical datasets analyzed during the current study are available in the FAOSTAT repository (http://www.fao.org/faostat/en/#data/QCL). Furthermore, the complete Python programming code (Google Colab notebook) and engineered feature datasets supporting the XGBoost and Score-Driven model implementations have been archived in a public repository [10.5281/zenodo.18776413] to ensure full methodological reproducibility.
